# Mucins: the frontline defence of the lung

**DOI:** 10.1042/BST20170402

**Published:** 2018-08-28

**Authors:** Caroline Ridley, David J. Thornton

**Affiliations:** Wellcome Trust Centre for Cell-Matrix Research, School of Biological Sciences, Faculty of Biology, Medicine and Health, Manchester Academic Health Sciences Centre, University of Manchester, Manchester M13 9PT, U.K.

**Keywords:** molecular basis of health and disease, mucin, mucus, respiratory physiology

## Abstract

Mucus plays a vital role in protecting the lungs from environmental factors, but conversely, in muco-obstructive airway disease, mucus becomes pathologic. In its protective role, mucus entraps microbes and particles removing them from the lungs via the co-ordinated beating of motile cilia. This mechanism of lung defence is reliant upon a flowing mucus gel, and the major macromolecular components that determine the rheological properties of mucus are the polymeric mucins, MUC5AC and MUC5B. These large O-linked glycoproteins have direct roles in maintaining lung homeostasis. MUC5B is essential for interaction with the ciliary clearance system and MUC5AC is up-regulated in response to allergic inflammatory challenge. Mucus with abnormal biophysical properties is a feature of muco-obstructive respiratory disease and can result from many different mechanisms including alterations in mucin polymer assembly, mucin concentration and the macromolecular form in mucus, as well as changes in airway surface hydration, pH and ion composition. The abnormal mucus results in defective lung protection via compromised ciliary clearance, leading to infection and inflammation.

## Introduction

The human lungs inhale up to 12 000 l of air a day, containing over 100 billion particles, chemicals and pathogens [1], and to maintain a healthy lung, the airways are protected by an extracellular hydrogel, the mucus barrier. Mucus forms a discontinuous layer and the maintenance of this barrier is vital for protection of the airway epithelium as it provides the first line of innate immune defence, trapping inhaled particles and pathogens, and dissolving noxious gases, thus preventing invasion and damage of the underlying tissue. The mucus-entrapped particles and toxicants are removed from the airways by the co-ordinated beating of cilia (mucociliary clearance, MCC), and impairment of this crucial protective mechanism results in mucus accumulation and plugging of the airways and infection and inflammation, which are common features of obstructive airway disease. Mucus is a complex, viscoelastic secretion containing proteins, lipids, ions and water; the major macromolecular components of mucus are the O-linked glycoproteins, mucins.

## Mucins

A total of 21 mucin genes have been identified (https://www.genenames.org), which are divided into two families: the secreted mucins and the cell-tethered mucins. The major mucins produced in the airways are the secreted polymeric mucins MUC5AC and MUC5B [2] and the cell-tethered mucins MUC1, MUC4, MUC16 and MUC20 [3]. The cell-tethered mucins form the basis of a gel-like layer surrounding the cilia (periciliary layer, PCL) that is essential for normal ciliary action to move mucus out of the airways [4]; whereas, the polymeric mucins, which are the major focus of this brief review, underpin the structure and organisation of the airways mucus gel and dictate its viscoelastic properties. In the upper airways, MUC5AC is mainly produced in epithelial surface goblet cells, whereas MUC5B is mainly secreted from mucous cells in submucosal glands [2,5,6]. However, in the more distal airways, both MUC5AC and MUC5B are produced from secretory cells in the epithelial surface [6–8].

## Polymeric airway mucins

**Figure 1.**
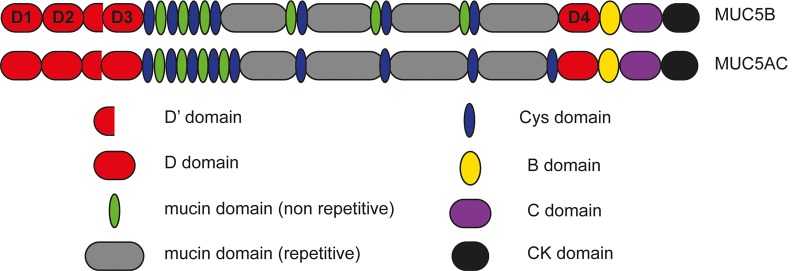
Schematic representation of the domain structure of the polymeric mucins MUC5AC and MUC5B. MUC5AC and MUC5B consist of vWF-like D domains (red) in the order D1–D2–D′–D3 at the N-terminus. The domains at the C-terminus are in the order D4–B–C–CK. The central mucin domain is composed of repetitive (grey) and non-repetitive sequences (green) enriched in serine, threonine and proline, interspersed with a variable number of Cys domains (blue); nine for MUC5AC and seven for MUC5B. The D3 and CK domains are the sites of intermolecular disulfide linkage that form the mucin polymers.

MUC5AC and MUC5B are large (5–50 MDa), heavily O-glycosylated proteins with characteristic structural domains (Figure 1). The N- and C-terminal regions consist of von Willebrand factor-like (vWF) domains in the order D1–D2–D′–D3 and D4–B–C–CK, respectively, which are involved in polymerisation. The central mucin domain is dominated by proline-, serine- and threonine-rich repetitive and non-repetitive sequences that undergo O-glycosylation and account for up to 80% of the mucin molecular mass [2,9]. While sharing common glycans, MUC5AC is more fucosylated, whereas MUC5B is more sialylated [10], and the extensive O-glycosylation results in a stiffened mucin backbone, important for gel formation [11]. The mucin glycans also play a central role in host–pathogen interactions, and alteration in the pattern of mucin glycans may lead to bacterial colonisation and infection, although there are conflicting reports regarding mucin glycosylation and bacterial interactions [9,12–15]. MUC5AC shares a similar glycosylation profile between individuals [16,17]. In contrast, MUC5B has different charged forms (low- and high-charge glycoforms), most probably due to different levels of sulfation; the high-charged glycoform being predominant in secretions from healthy airways [16]. The central mucin domain is interspersed with a variable number of cysteine-rich (Cys-rich) domains; there are nine and seven Cys domains present in MUC5AC and MUC5B, respectively. These domains are highly conserved and may have a role in controlling the network properties of mucus via non-covalent cross-links between mucin polymers [18,19].

MUC5B and MUC5AC have distinct roles in maintaining a healthy airway epithelium. MUC5B is the predominant mucin in healthy human airways [16,17,20], and studies in mice deficient in Muc5b production have shown that Muc5b is essential for MCC and the maintenance of a healthy airway by controlling bacterial infection and resolving inflammation following infection [21]. In contrast, MUC5AC production is much lower in healthy airways, but is up-regulated, for example, in response to viral infections, where it acts as a decoy for viral receptors and is essential for an inflammatory response [22–24]. In contrast with Muc5b, mice deficient in Muc5ac survive normally, with no impairment in MCC or immune homeostasis of the lung [21].

## From polymeric mucins to mucus

### Polymeric mucin assembly

Owing to their complex nature and extreme size, mucins undergo a multi-step synthesis, with some mechanistic details still remaining unclear. Here, we will outline the current knowledge of polymeric mucin assembly that pertains to both MUC5AC and MUC5B. The assembly of the polymer takes ∼2 h. The fully assembled polymers are stored within intracellular storage granules and the majority of mucins are released by 48 h [25,26]. In the endoplasmic reticulum, the mucin molecule undergoes N-glycosylation and C-mannosylation, and forms disulfide-linked dimers via the CK domain in the C-terminus [25,27]. The dimers transit the Golgi where they undergo extensive O-glycosylation, then further polymerisation via disulfide linkage between D3 domains in the N-terminus, to form linear polymers [26,28–30].

### Intragranular storage

Prior to secretion, the mucin polymers are compacted, dehydrated and stored in secretory granules, at a low pH (∼pH 5.5) in the presence of calcium ions, and it is well established that calcium ions shield the anionic charge on the sialic acid and sulfate groups on mucin glycans, aiding mucin compaction [31,32]. In addition, calcium ions have specific interaction with the mucin polypeptide [33]. Non-covalent, calcium-dependent interactions between the dimeric N-terminal domains of MUC5B (within and/or between polymers) are active at the acidic conditions of the late secretory pathway and act to organise the mucin chains within the secretory granules [28,34].

### Post-secretory expansion

Upon granular release, the pH and calcium-dependent N-terminal intramolecular interactions are uncoupled and the glycan-associated calcium ions are replaced by sodium ions [35]. This leads to an increase in mucin hydration and its rapid expansion and change in conformation from a compact to a linear form, which is required for the correct rheological properties of the mucus gel [31,34]. Mucin expansion is dependent on many factors including airway surface hydration, pH and $\mathrm{HC}O_{3}^{-}$ availability [36–40], with a suboptimal extracellular environment leading to aberrant mucus formation.

### Mucins in mucus

**Figure 2.**
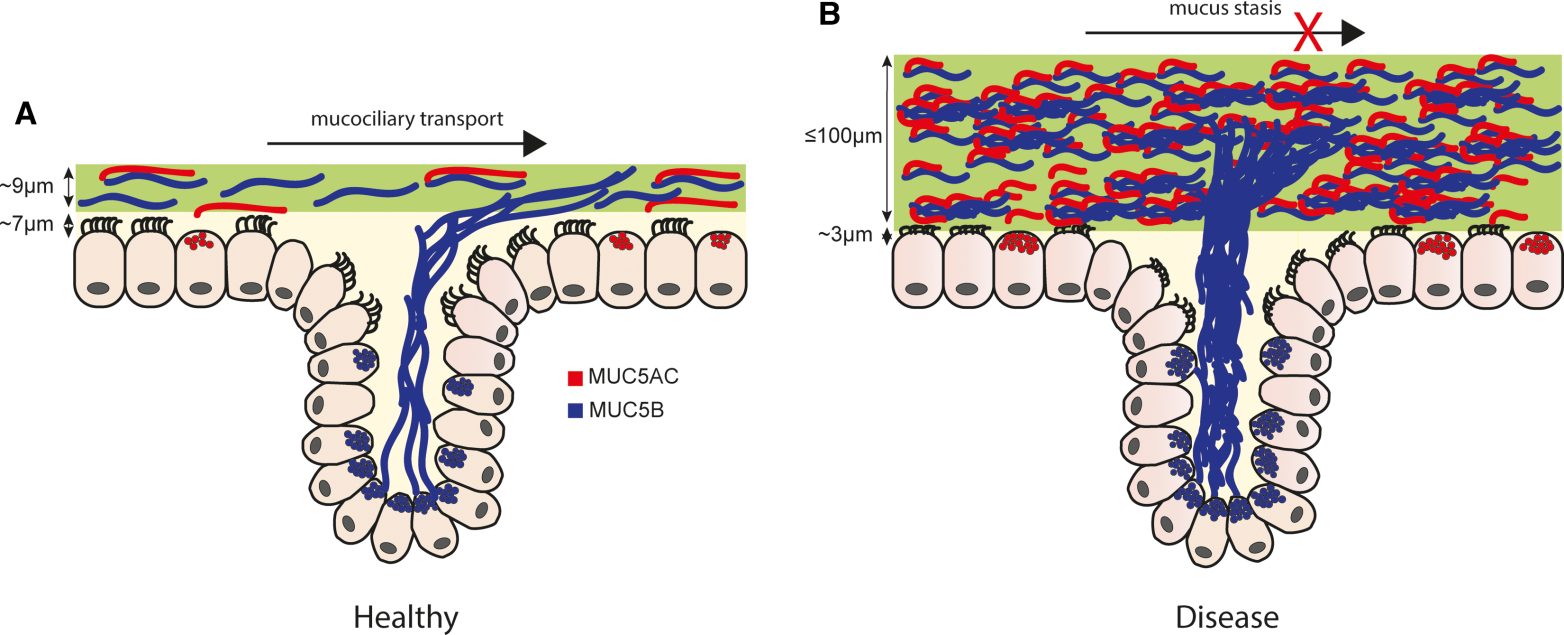
Lung mucins in health and disease. (**A**) In healthy airways, the protective mucus barrier consists mainly of MUC5B (blue) with a lesser amount of MUC5AC (red) that entraps inhaled particles and microbes. MUC5B bundles are shown emanating from submucosal glands. Optimal airway hydration maintains the correct rheological properties of the mucus gel, permitting efficient MCC. (**B**) In obstructive airway disease, mucin concentration is increased, which collapses the PCL, compresses cilia and impairs or stops MCC.

The macromolecular form and organisation of MUC5AC and MUC5B polymers within mucus are still open questions, since much of our knowledge of mucin structure is based on information gained from studies on purified mucins. Immunolocalisation of MUC5AC and MUC5B suggests that these two mucins are spatially separated in mucus [20,41,42]. Recent studies in pigs have reported that MUC5B is released from submucosal gland ducts in the form of strands or ‘mucin bundles’ between 5 and 50 µm in diameter, extending up to hundreds of micrometres in length [43–46] (Figure 2). These bundles probably represent the side-by-side association of multiple mucin chains. In contrast, MUC5AC, released from surface goblet cells, appeared as ‘wispy threads and sheets’, between 1 and 4 µm in diameter [45]. However, once on the surface epithelium, the MUC5B strands can become partially coated with MUC5AC [20,41–46]. Whether MUC5B produced from epithelial surface secretory cells associates laterally to form bundles is not known. Understanding the functional significance of these different structural morphologies and if they are influenced by components of the mucin interactome [47] requires further research.

## The airway mucin barriers

In the airways, there are two distinct mucin-rich environments: the PCL and the viscoelastic mucus gel. The PCL is occupied by a high concentration of cell-tethered mucins; MUC16, MUC4 and MUC20 are localised to the cilia and MUC1 is located on microvilli, with mucin lengths extending between 190 and 1500 nm [4,42]. The intermolecular repulsion between the polyanionic cell-tethered mucins forms a semipermeable, brush-like barrier, with a maximum pore size of ∼40 nm, protecting the airway epithelium from virus penetration [4,48,49]. This mucin-rich environment also plays a critical role in regulating the movement of the mucus by cilia. In brief, the intermolecular repulsion of the cell-tethered mucins generates an osmotic pressure in the PCL (∼300 Pa) that is higher than that generated by the overlying mucus gel (∼200 Pa), which keeps the mucus layer above the cilia. An optimal PCL height for MCC is 5–8 µm, and this is maintained through regulated airway surface hydration, permitting free movement of the cilia and optimal MCC [4,42,50].

## Polymeric mucins in airway diseases

Muco-obstructive airway diseases have common features, including mucin-producing cell hyperplasia, mucin hypersecretion and an altered mucin macromolecular form, which contribute to the formation of a dysfunctional mucus gel. As a consequence, mucus accumulates in the airways causing obstruction which results in infection and inflammation. Mucus with aberrant properties that compromises airway clearance (Figure 2) may be caused by many different mechanisms: for example, defective mucin intracellular assembly and intragranular packaging, defective post-secretory mucin expansion [34,36,38,42,51,52] as a result of a non-optimal local environment (hydration, pH and $\mathrm{HC}O_{3}^{-}$) [4,37,53,54], formation of permanent cross-links between mucin chains [55–57], alterations in the relative amounts of MUC5AC and MUC5B, and/or the different glycosylated variants of MUC5B [17,20,41,56] and increased mucin concentration [4,58,59]. In the next section, we will briefly review the evidence linking changes in mucin concentration, type and macromolecular form to the formation of dysfunctional mucus in airway disease.

### Mucin concentration

For effective barrier protection, mucus ideally comprises 98% water and 2% solids (predominantly mucins). However, in muco-obstructive disease [asthma, cystic fibrosis (CF) or chronic obstructive pulmonary disease (COPD)], the mucin content of mucus increases (3–9% [60]), which, coupled with a reduction in water content to ≥90%, results in impaired MCC [61–63]. In these obstructive airway diseases, one mechanism by which increased mucin concentration impairs MCC is by increasing the osmotic pressure of the mucus layer which compresses the PCL and collapses the cilia [4]. The static mucus barrier transforms from being protective to pathological, harbouring bacteria and resulting in infection and inflammation [64–67]. It is clear that mucin concentration is critical for effective mucus transport, with increased mucin concentration resulting in increased mucus viscoelasticity and dysfunctional MCC [58,68]. However, in disease, other components augment mucus viscoelasticity, for example, actin and DNA [69–71]. Thus, there is no simple relationship between mucin concentration and mucus viscoelasticity that might predict disease severity.

### Mucin type — gene products and glycoforms

In CF and COPD, MUC5B is the predominant mucin in the material expectorated from the lungs (sputum). In COPD sputum, both MUC5AC and MUC5B are increased; there is an increased ratio of MUC5AC to MUC5B, with MUC5AC levels elevated in smokers with and without COPD [16,72,73]. MUC5B overproduction correlates with an increase in disease severity and decreased lung function [72,73]. In asthma, MUC5AC levels are significantly increased, whereas MUC5B levels remain stable or decrease, compared with healthy lungs [16,17,20,24,74,75]. Interestingly, in asthma, MUC5AC has been shown to tether to the epithelium, which has been implicated in mucociliary dysfunction and mucus plug formation [41].

The increase in MUC5B observed in CF and COPD is mainly due to an increase in the low-charge glycoform [16,73]. Moreover, the low-charge glycoform of MUC5B was the prominent mucin in the sputum from smokers with airways obstruction. In fatal asthma, the low-charge glycoform of MUC5B was enriched in the viscid mucus plugging the airways [76]. More recently, analysis of MUC5B glycoforms in children with asthma showed that the low-charge glycoform was most associated with acute asthma [17]. These studies generally show an increase in this glycosylated variant of MUC5B in disease; however, the functional significance of different MUC5B glycoforms remains to be elucidated.

The nature of the difference in mucin charge glycoforms has not been determined. However, the non-reducing terminal structures on mucin glycans found on MUC5AC and MUC5B contain both sulfate groups and sialic acid (with and without O-acetylation) residues [77–82]. Furthermore, mucins are substituted with a diverse array of *O*-glycans which can vary extensively between individuals and this is in part influenced by the expression of blood group antigens, Lewis group antigens and secretor status, with MUC5B being a predominant carrier of these antigens [83–85]. Individuals are classed as secretors or non-secretors of blood group antigens, with secretors accounting for ∼80% of Caucasians. Secretor status appears to be protective for certain bacterial and fungal infections [83], but is associated with a susceptibility to respiratory viruses [86], whereas non-secretors express more sialylation [85] and are more susceptible to bacterial and fungal infections.

### Mucin macromolecular form

Alteration in mucin macromolecular form has been suggested to play an important role in the formation of mucus gels with aberrant protective function [55,56,72]. This may arise due to the formation of inappropriate cross-links between mucin polymers in mucus and/or failure to uncouple the mucin cross-links (post-secretion) required for intragranular packaging. In fatal asthma, MUCB (low-charge glycoform) was shown to have unusual morphology and was highly cross-linked compared with the linear architecture of mucins isolated from healthy airway sections [30,76]. At the time, it was hypothesised that MUC5B had not undergone proteolytic processing to produce the linear chains observed for airway mucins by electron microscopy [30,34,76]. However, recent evidence suggests that MUC5B does not undergo proteolytic processing as part of its assembly process [25], and that reactive oxygen species on the lung epithelium generate thiol cross-links between mucin chains that are probably responsible for abnormal mucin morphology and mucus plug formation [55].

An altered airway surface environment as seen in CF (decreased Cl^−^ and $\mathrm{HC}O_{3}^{-}$ concentration, lowered airway surface pH and dehydration [52,87,88]) may cause failure in the uncoupling of mucin polymer interactions that are active in intragranular packaging, thus negatively affecting the transition from the non-covalently, cross-linked intragranular form to the more expanded linear architecture in mucus [28,72]. By whatever mechanism, the abnormal macromolecular form of mucin polymers will probably result in mucus with aberrant biophysical properties that will contribute to dysfunction in MCC. One obvious target to normalise mucus properties is to disrupt the disulfide linkages responsible for mucin polymer formation and for cross-linking between mucin chains. Currently, *N*-acetylcysteine is employed to target these linkages in mucins and thus act as a mucus-solubilising agent (mucolytic). However, its efficacy is limited [89], and more effective and potent mucolytics are being developed [57,90].

## Summary

The polymeric mucins MUC5AC and MUC5B play a vital role in protection of the lungs. Understanding of lung mucin structure and function has advanced greatly in the past decade, but there are still many fundamental gaps in our knowledge. For example, what is the supramolecular organisation of a mucin polymer in mucus? How are mucin protective properties modulated by other components of mucus? Do the different glycosylated variants of MUC5B have different functional properties? Are particular glycan structures linked with normal and pathological mucin expression in disease? Most importantly, what are the critical factors controlling mucus gel formation. Improved knowledge of these basic aspects of mucin biology will inform strategies to alleviate mucus obstruction of the airways.

## Abbreviations

CF: cystic fibrosis
COPD: chronic obstructive pulmonary disease
MCC: mucociliary clearance
PCL: periciliary layer
vWF: von Willebrand factor.
